# Animal model of arthritis and myositis induced by the Mayaro virus

**DOI:** 10.1371/journal.pntd.0007375

**Published:** 2019-05-03

**Authors:** Franciele Martins Santos, Roberto Sousa Dias, Michelle Dias de Oliveira, Isabella Cristina Toledo Alves Costa, Luciana de Souza Fernandes, Carine Ribeiro Pessoa, Sérgio Luis Pinto da Matta, Vivian Vasconcelos Costa, Danielle G. Souza, Cynthia Canêdo da Silva, Sérgio Oliveira de Paula

**Affiliations:** 1 Molecular Immunovirology Laboratory, Department of General Biology, Federal University of Viçosa, Viçosa, Minas Gerais, Brazil; 2 Structural Biology Laboratory, Department of General Biology, Federal University of Viçosa, Viçosa, Minas Gerais, Brazil; 3 Morphology Department, Federal University of Minas Gerais, Belo Horizonte, Minas Gerais, Brazil; 4 Department of Microbiology, Federal University of Minas Gerais, Belo Horizonte, Minas Gerais, Brazil; 5 Department of Microbiology, Federal University of Viçosa, Viçosa, Minas Gerais, Brazil; University of Pittsburgh, UNITED STATES

## Abstract

**Background:**

The Mayaro virus (MAYV) is an endemic arbovirus in South American countries, where it is responsible for sporadic outbreaks of Mayaro fever. Clinical manifestations include fever, headache, ocular pain, rash, myalgia, and debilitating and persistent polyarthralgia. Understanding the mechanisms associated with MAYV-induced arthritis is of great importance due to the potential for its emergence, urbanization and dispersion to other regions.

**Methods:**

15-day old Balb/c mice were infected by two distinct pathways, below the forelimb and in the rear footpad. Animals were observed for a period of 21 days. During this time, they were monitored every 24 hours for disease signs, such as weight loss and muscle weakness. Histological damage in the muscles and joints was evaluated 3, 7, 10, 15 and 20 days post-infection. The cytokine profile in serum and muscles during MAYV infection was evaluated by flow cytometry at different post-infection times. For pain analysis, the animals were submitted to the von Frey test and titre in different organs was evaluated throughout the study to obtain viral kinetics.

**Findings:**

Infection by two distinct pathways, below the forelimb and in the rear footpad, resulted in a homogeneous viral spread and the development of acute disease in animals. Clinical signs were observed such as ruffled fur, hunched posture, eye irritation and slight gait alteration. In the physical test, both groups presented loss of resistance, which was associated with histopathological damage, including myositis, arthritis, tenosynovitis and periostitis. The immune response was characterized by a strong inflammatory response mediated by the cytokines TNF-α, IL-6 and INF-γ and chemokine MCP-1, followed by the action of IL-10 and IL-4 cytokines.

**Interpretation:**

The results showed that Balb/c mice represent a promising model to study mechanisms involved in MAYV pathogenesis and for future antiviral testing.

## Author summary

The Mayaro virus, although restricted to some regions of Latin America, has great potential for emergence, which makes it of great medical-scientific interest. Therefore, pathogenesis study of the MAYV in an animal model has fundamental importance for the determination of viral and host factors that contribute to disease development. In addition, it will allow develop and evaluate the effectiveness of possible antiviral agents. In this study, the authors were able to standardize a disease model for the MAYV in BALB / c mice. From the obtained data it is possible to observe the induction of acute arthritis and myositis, accompanied by the reduction of physical strength of the animals. As described for other alphaviruses in both animal models and patient, the proinflammatory mediators TNF-α, IL-6, INF-γ and MCP-1 were elevated in the serum of MAYV-infected animals and therefore appeared to be mediators which also play an important role in the pathogenesis of MAYV.

## Introduction

Mayaro virus (MAYV, genus *Alphavirus*, *Togaviridae* family) is an emergent arbovirus, responsible for sporadic cases, outbreaks and small epidemics of acute febrile disease in South American countries, particularly in the Amazon basin. However, serological data have shown the circulation of the MAYV in Central American countries, such as Guatemala, Costa Rica, Panama and more recently in Haiti, where a new strain was isolated [1,2]. MAYV infections mainly affect people living or working near forest areas where MAYV is kept in an enzootic cycle, which primarily involves the *Haemagogus janthinomys* mosquito as a vector and nonhuman primates as natural hosts, however other vectors (*Culex sp*, *Sabethes sp*, *Psorophora sp*, *Coquillettidia sp* and *Aedes sp* mosquitoes) and wild vertebrates (marsupials, rodents and birds) may be important in the transmission cycle and spread of the virus [3].

The MAYV as well as the chikungunya virus present a high potential for urbanization and emergence due to the ability of these viruses to mutate and adapt to new transmission cycles [4]. Recent studies in Brazil have already demonstrated the occurrence of Mayaro fever in urban areas of Manaus city and Mato Grosso state [5,6,7]. In Cuiabá, capital of Mato Grosso state, an entomological surveillance study identified the species of *Culex quinquefasciatus* and *Aedes aegypti* as mosquitoes naturally infected by MAYV, corroborating with the occurrence of urban transmission in Cuiabá and possibly in other cities of central region of Brazil [7]. This shows that Mayaro fever should be included in the differential diagnosis with dengue virus (DENV), chikungunya virus (CHIKV) and Zika virus (ZIKV) in areas where there is co-circulation of these arboviruses [8]. The recent MAYV case in Haiti poses a potential threat for spreading through Central and North America. Due to its geographical proximity, vector species and population flows, the United States is the country with the highest risk for MAYV emergence [9,10]. In addition, reports of European tourists who visited the Amazon region and returned to their countries with symptoms characteristic of Mayaro fever, highlights concerns regarding MAYV as a potential emerging disease in Europe, where *Aedes albopictus* is present and experimentally able to transmit the MAYV [11,12].

Mayaro fever is characterized as a self-limiting disease, which can range from mild to moderately severe. Clinical manifestations include fever, headache, ocular pain, rash, photophobia, joint edema, myalgia, and arthralgia. Hemorrhagic phenomena such as petechiae, gingival bleeding and epistaxis have also been observed [5]. Like other arthritogenic alphaviruses, MAYV also induces disabling and long-lasting polyarthralgias, with the joints of the wrist, ankle and small joints of hands and feet being predominantly affected [13].

As the pathophysiology of the MAYV is not fully elucidated and represents an important public health issue, the objective of this research was to establish an animal model for the study of the pathogenesis induced by the MAYV. The results demonstrated that Balb/c mice at 15-days developed acute disease with musculoskeletal damage, which included myositis, arthritis, tenosynovitis and periostitis. A strong inflammatory response was induced on initial days of infection, which was controlled through the participation of IL-4 and IL-10 cytokines. These results present an animal model to study the pathogenesis of the Mayaro virus.

## Results

### Assessment of clinical signs of the disease in infected animals

**Fig 1:**
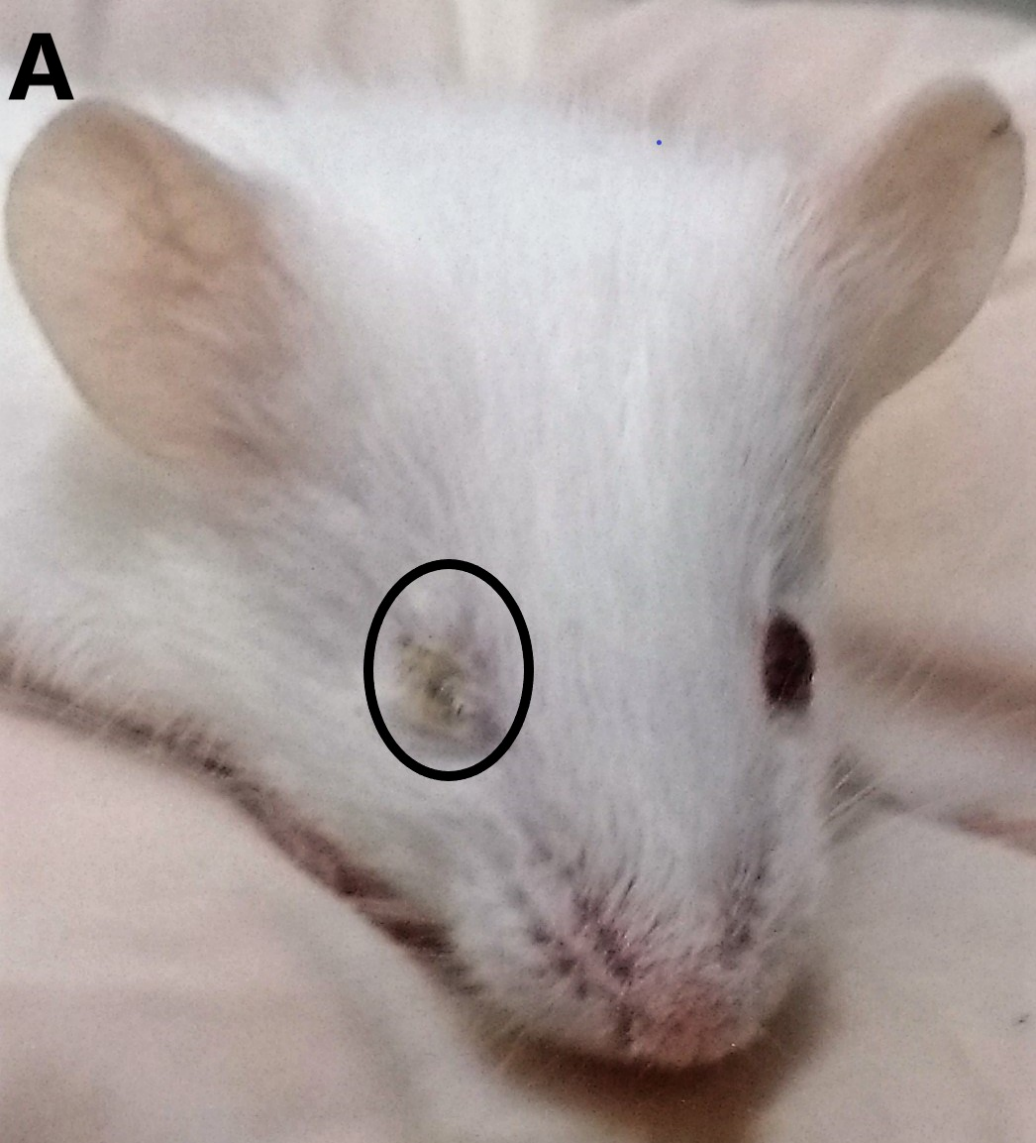
Balb/c mice with MAYV infected below the forelimb. Animals showing eye irritation and ruffled fur at 8 days post-infection. The irritation in the eye presented unilaterally, with tearing.

**Fig 2:**
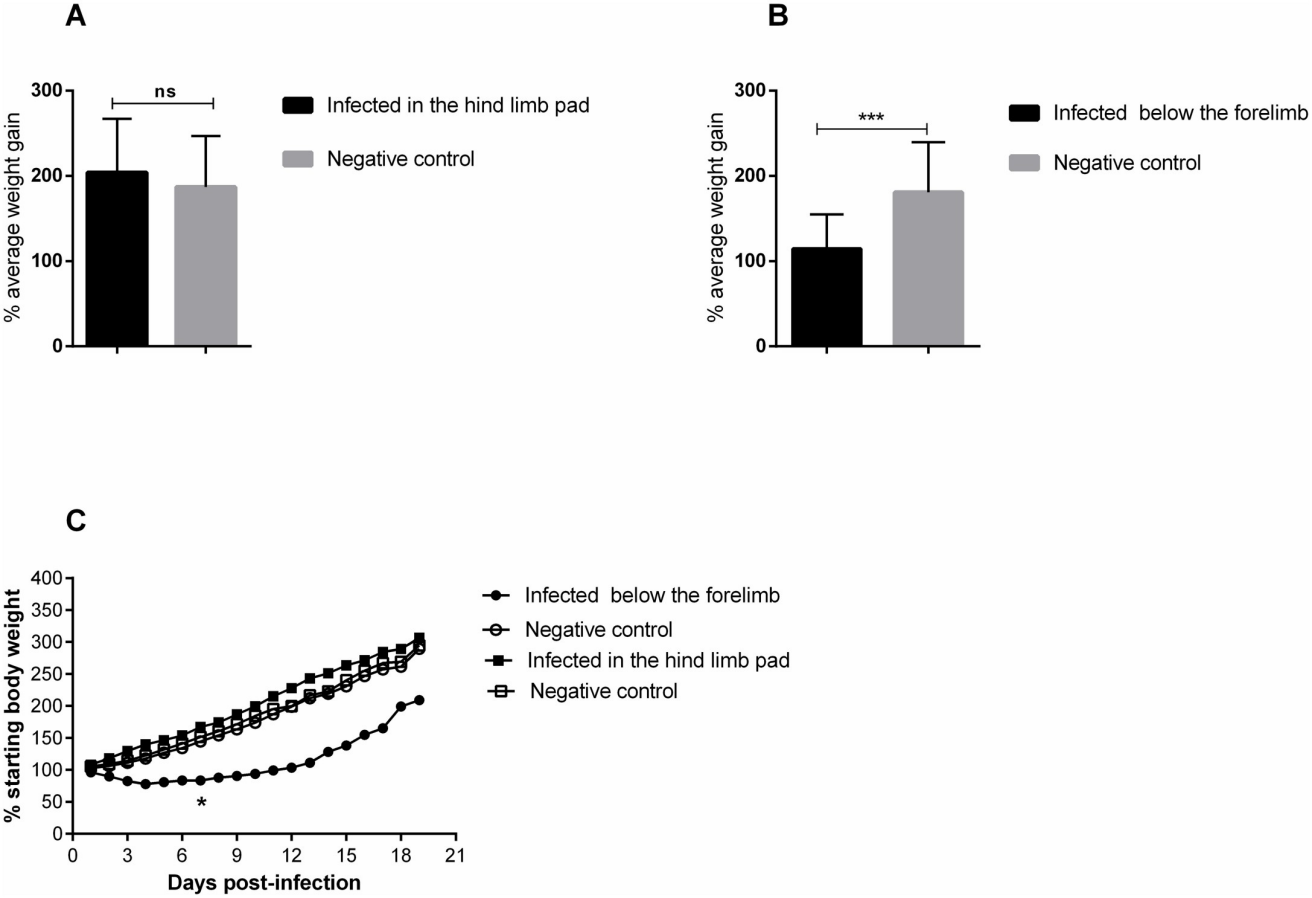
Mayaro virus induces weight loss in 15-day old Balb/c mice. Mice weight was evaluated throughout the experiment. 15-day old Balb/c mice were infected with 2.57 x 10^6^ PFU in the rear footpad and 1.25 x 10^7^ PFU below the forelimb by subcutaneous injection. A) Infected mice did not present a statistical difference in hind limb pad pathway. B) A statistical difference was observed for the below the forelimb pathway. C) Behavior of the groups throughout the experiment. Control groups received only PBS 1X. The animals were monitored at 24-hour intervals for weight gain or loss, and statistics were ascertained with two-way ANOVA, Tukey’s multiple comparisons test (* P value ≤ 0.05;*** P value <0.0002). N = 12 animals/group.

**Fig 3:**
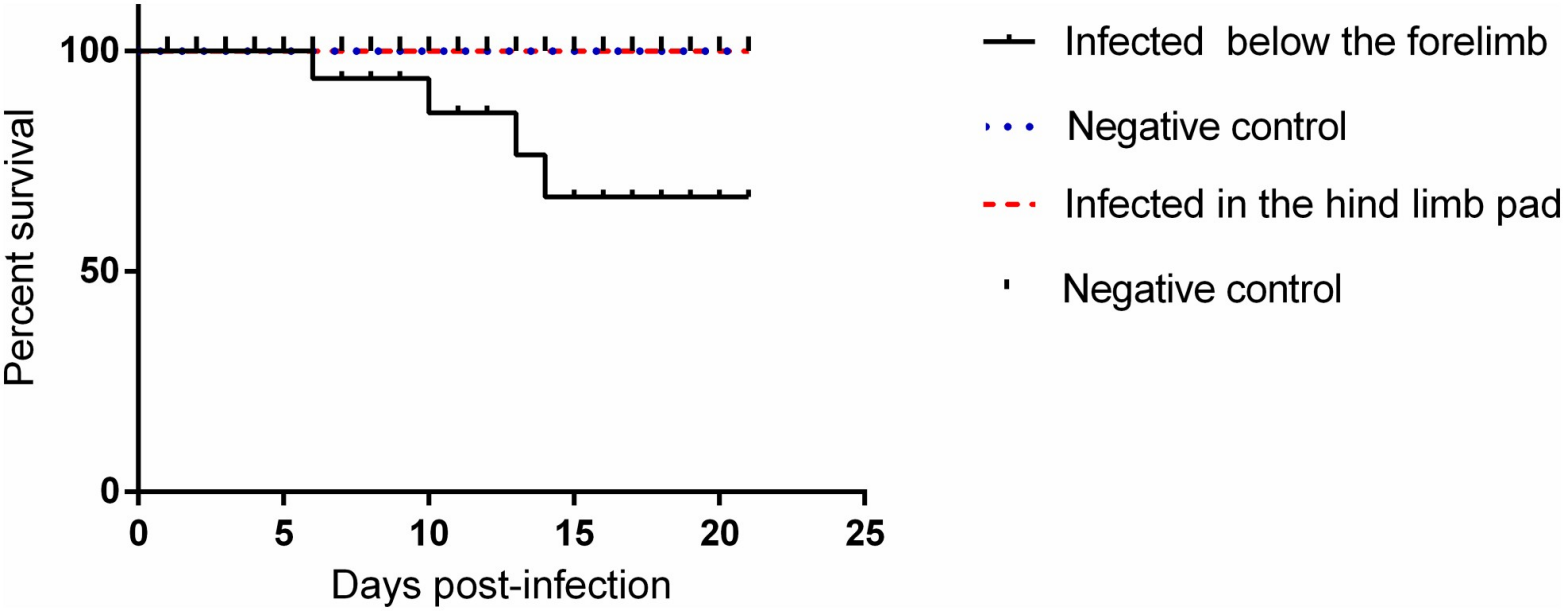
Survival of 15-day-old Balb/c mice infected below the forelimb subcutaneously. (N = 12 animals/group).

The inoculation of the animals was performed via two distinct pathways, below the forelimb and in the rear footpad, determined as FLP and FPP, respectively. Clinical signs such as ruffled fur, hunched posture and eye irritation were observed in both groups (Table 1). Eye irritation was characterized by tearing, blepharospasm, and may present unilaterally or bilaterally (Fig 1). Only FLP animals presented weight loss and gait alteration (Fig 2A, 2B and 2C). Weight loss in FLP begins at day 1 PI, while in the other groups, we observed a gradual increase in body weight. A significant difference between FLP and the control group is evident on day 7 PI and remains up to day 19 PI. Some signs such as hind limb drag, tremors, and falling and circulating spontaneously were observed in a single experiment in the FLP animals. The change in the animals′ gait was more pronounced from day 10 PI. Tremors were observed only in three animals and only one animal showed the signal falling and circulating spontaneously. In addition, 33% of the animals succumbed to infection in the same experiment (Fig 3).

**Table 1:** Clinical signs observed in Balb / c mice during virus infection Mayaro.

	Infected below forelimb		Infected in the hind limb pad	
	Days PI	N° of animals	Days PI	N° of animals
*Ruffled fur	2–21	12	2–20	10
*Hunched posture	2–19	12	2–19	10
*Eye irritation	variable	7	variable	2
*Gait alteration	6–12	9	----	8
**Tremors**	7, 20–21	2	----	0
**Moribund**	5–16	6	----	0
**Animals falling and circulating spontaneously**	7	1	----	0
**Death**	6, 8, 13, 14	4	----	0

* Common clinical signs of infection.

----Absence of clinical signs. PI: post-infection.

### Loss of physical strength

**Fig 4:**
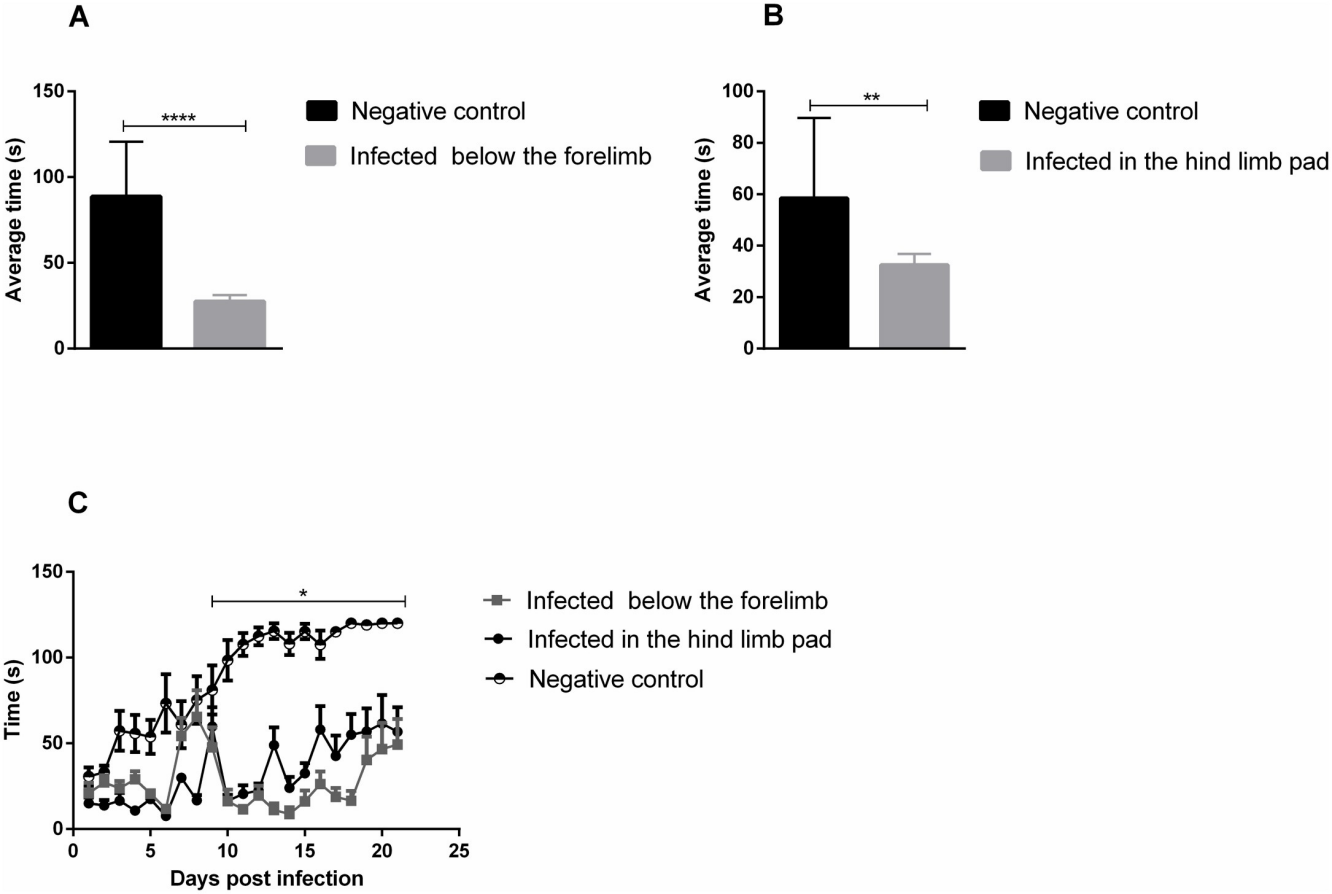
Evaluation of muscle weakness in 15-day-old Balb/c mice. Mice were subjected to the Wire-hang physical test at 24-hour intervals. 15-day old Balb/c mice were infected with 2.57 x 10^6^ PFU in the rear footpad and 1.25 x 10^7^ PFU below the forelimb by subcutaneous injection. Control groups received only PBS 1X. (A and B) average time that animals were able to remain attached to the grid, infected groups presented significantly lower means than the control time. (C) Behavior of the groups throughout the experiment. Statistics were performed with two-way ANOVA, Tukey’s multiple comparisons test. (* P value ≤ 0.05;** P value <0.0034; **** P value <0.0001). N = 12 animals/group.

In the physical test, it was found that both infected groups showed significant reduction in time differences when compared to controls (Fig 4A, 4B and 4C). In the Figure, it can be observed that in the first five days, all groups had very close and low time averages (Fig 4C). Up to the tenth day post-infection, an increase in the time of all four groups was observed. However, from the tenth day a significant difference was observed between the infected and uninfected groups, which continued up to day 19 PI. On day 16, FPP began to increase the permanence time adhered to the grid, while in the FLP animals, this increase occurred from day 17.

### Myositis and arthritis induced by the Mayaro virus

**Fig 5:**
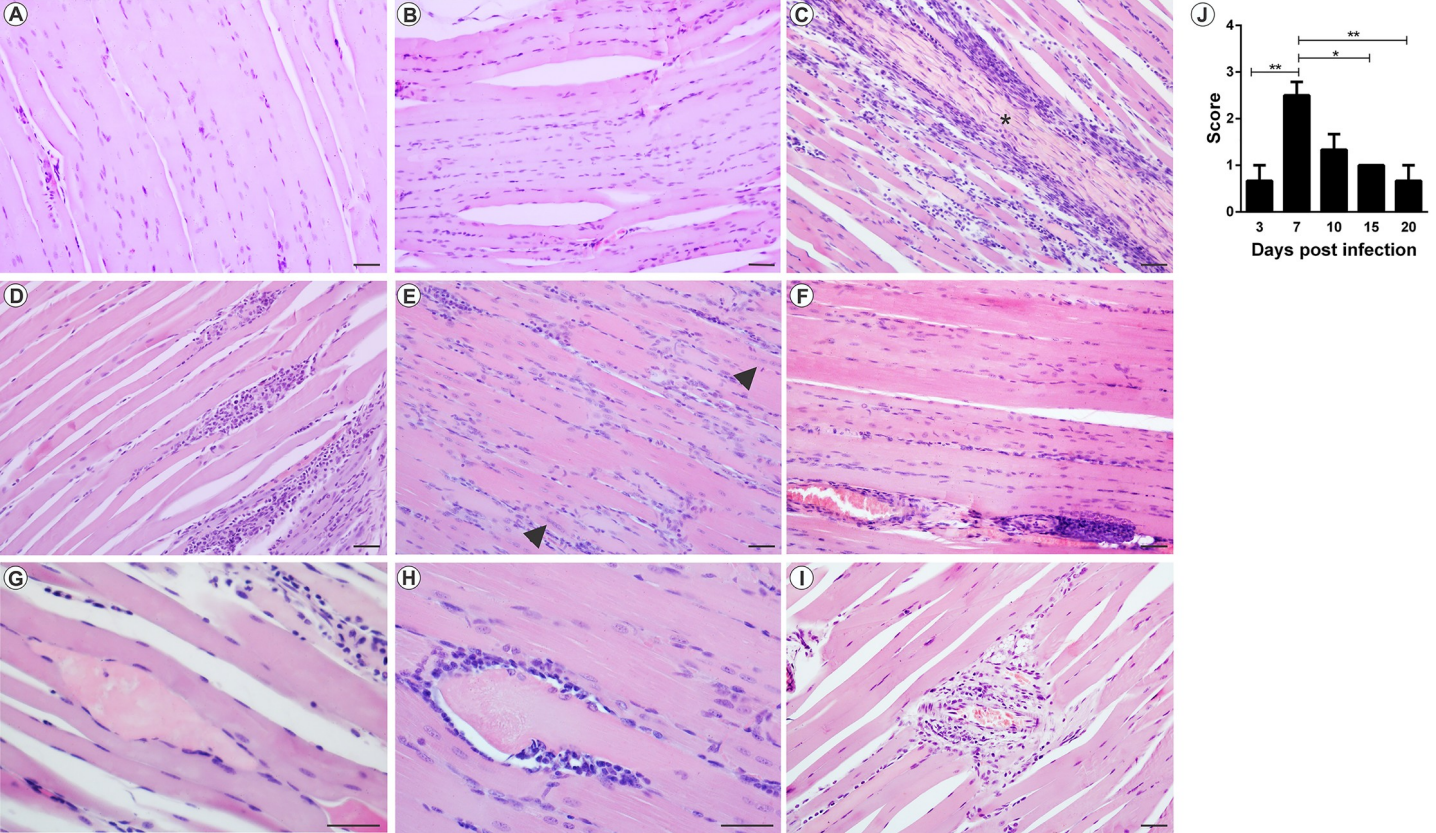
Mayaro virus-induced myositis in the ipsilateral muscle in mice infected below the right forelimb with 1.25x10^7^ PFU. The animals were sacrificed on days 3, 7, 10, 15 and 20 PI. for tissue damage analysis. The quadricep muscle was fixed in Karnovsky, embedded in paraffin, and 5μm sections were made and stained with HE. (A) Negative control. (B) MAYV infection at 3 days PI. (C) MAYV infection at 7 days PI (D) MAYV infection at 10 days PI (E) MAYV infection at 15 days PI. Fibers with centralized nuclei (arrowhead). (F) MAYV infection at 20 days PI. (G) Fiber in degeneration, with pale color and swollen appearance. (H) Region of degenerating fiber undergoing phagocytosis. (I) Vasculitis. (J) Histological score. Images A, B, C, D, E, F and I (200X magnification, 40 μm bar); G and H pictures (magnification 400X, bar 40 uM). N = 3 animals/group.

**Fig 6:**
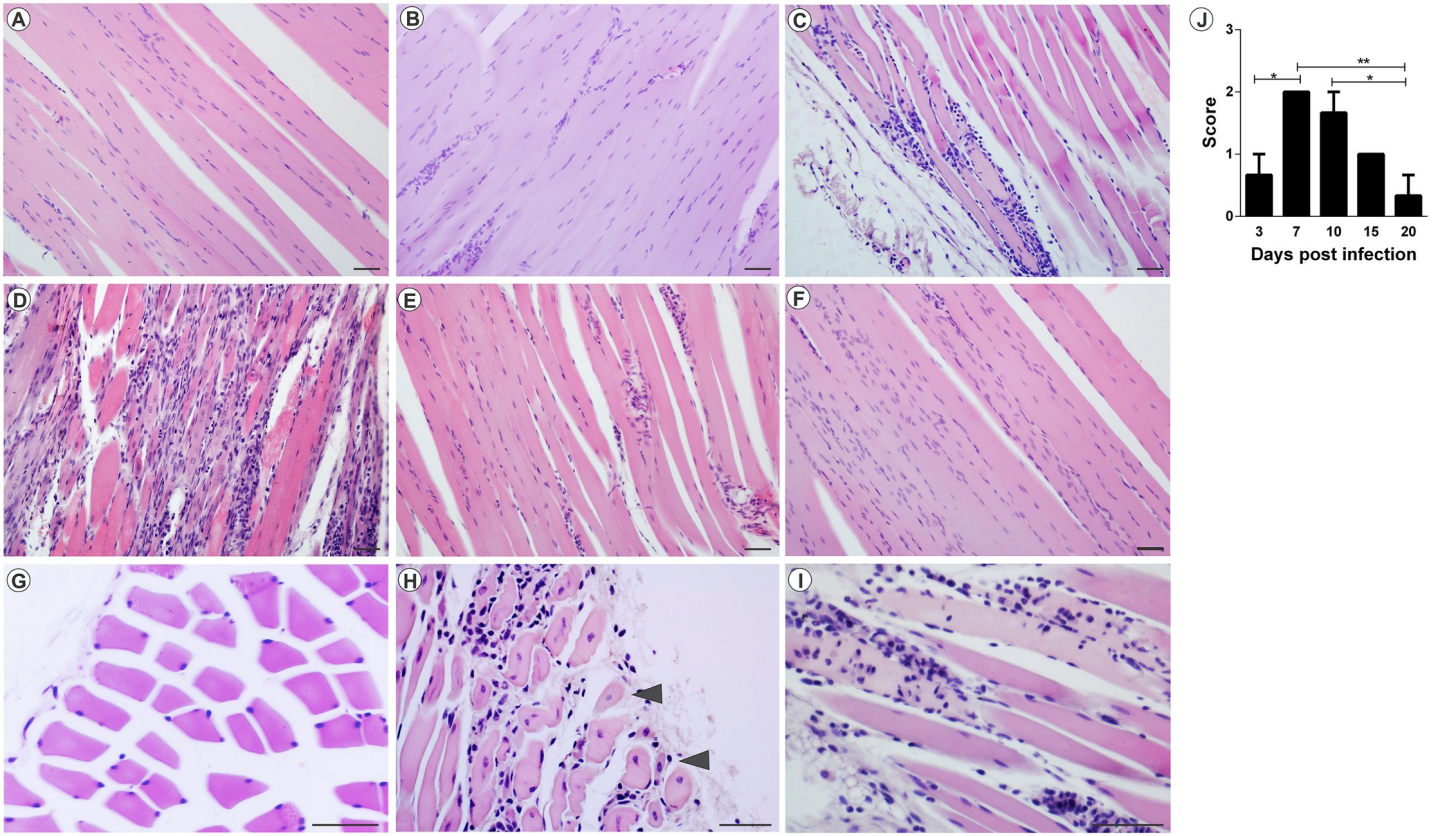
Myositis Mayaro virus-induced contralateral muscle in mice infected below the right forelimb with 1.25x10^7^ PFU. The animals were sacrificed on days 3, 7, 10, 15 and 20 PI. for tissue damage analysis. The quadricep muscles were fixed in Karnovsky, embedded in paraffin, and 5μm sections were made and stained with HE. (A) Negative control. (B) MAYV infection at 3 days PI. (C) MAYV infection at 7 days PI. (D) MAYV infection at 10 days PI. (E) MAYV infection at 15 days PI. (F) MAYV infection at 20 days PI. (G) Negative control, fibers in cross-section. (H) Fibers in cross section showing centralized nuclei (arrowhead). (I) Fibers undergoing phagocytosis. (J) Histological score. Images A, B, C, D, E, F (200X magnification), G, H and I images (400X magnification). 40μm bar. N = 3 animals/group.

**Fig 7:**
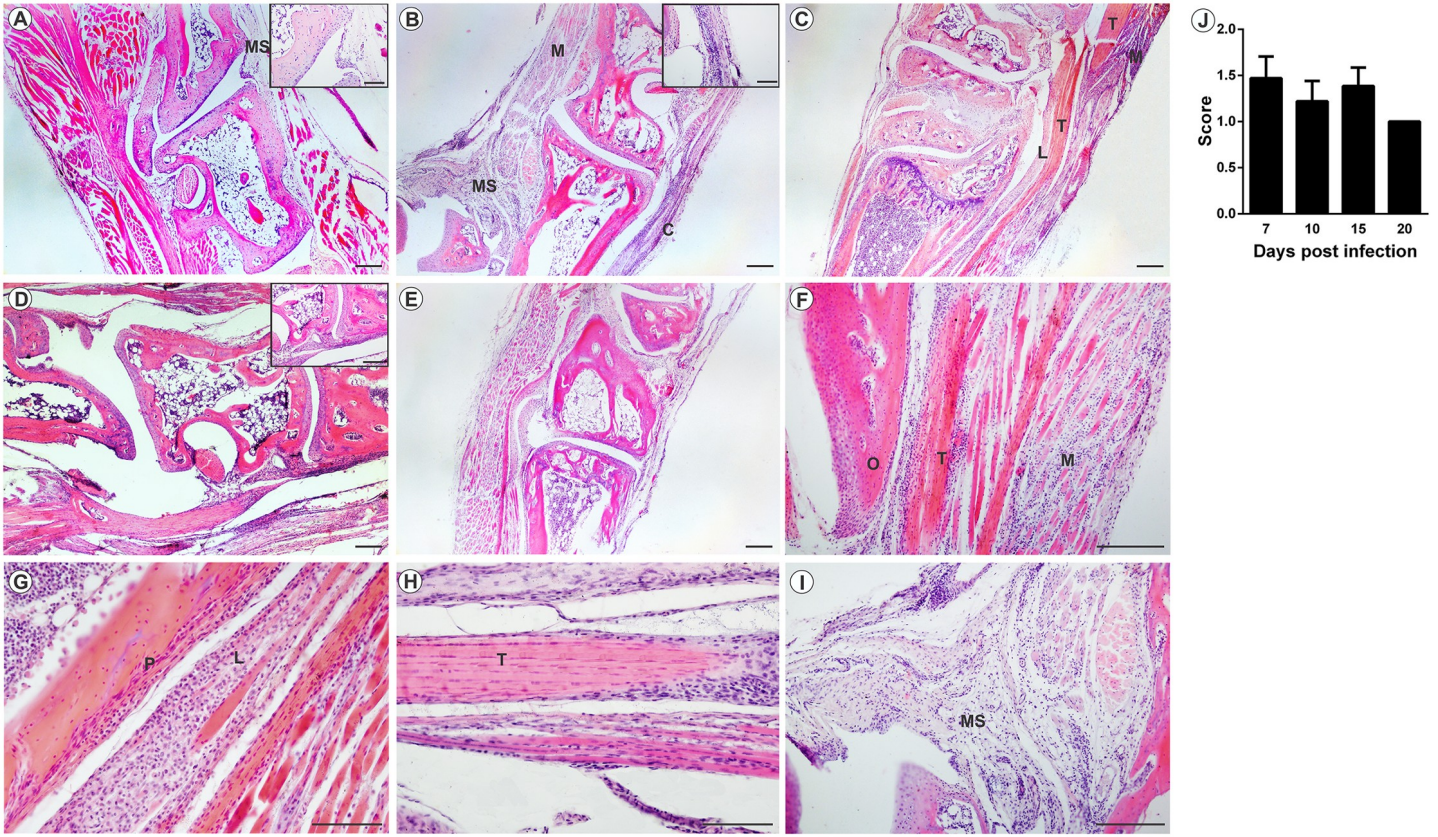
Mayaro virus-induced arthritis in mice infected below the right forelimb with 1.25x10^7^ PFU. Animals were sacrificed at 3, 7, 10, 15 and 20 days p.i. for tissue damage analysis. The ankle joint was fixed in Karnovsky, decalcified in 10% EDTA, embedded in paraffin, and 5μm sections were made and stained with HE. (A) Negative control. (B) Infiltrated in the joint capsule, muscle and synovial membrane with 7 days PI. (C) Infiltrated the ligament, tendon and muscle with 10 days PI. (D) Infiltrated synovial membrane with 15 days PI. (E) MAYV infection with 20 days PI. (F) Tendonitis and myositis with 7 days PI. (G) Periostitis with 10 days PI. (H) Tenosynovitis with 15 days PI. (I) Synovitis with 7 days PI. (J) Histological score. M: muscle; T: tendon; L: ligament; B: bone; P: periosteum; SM: Synovial membrane. Images A, B, C, D, E (40X magnification, 200 μm bar); F and I images (100X magnification), G and H images (200X magnification) 100 μm bar. N = 3 animals/group.

**Fig 8:**
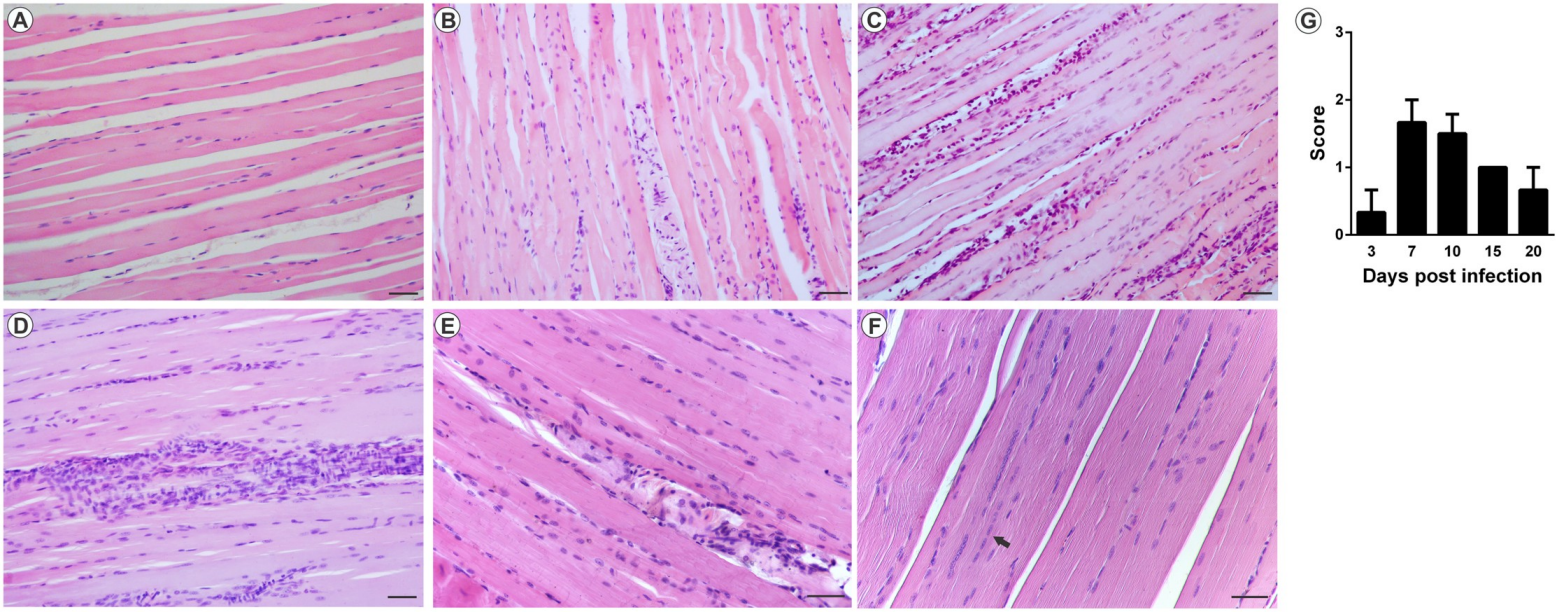
Mayaro virus-induced myositis in the ipsilateral muscle in mice infected via the rear left footpad with 2.57 x 10^6^ PFU. Animals were sacrificed on days 3, 7, 10, 15 and 20 PI. for tissue damage analysis. The quadricep muscle was fixed in Karnovsky, embedded in paraffin, and 5μm sections were made and stained with HE. (A) Negative control. (B) MAYV infection at 3 days PI. (C) MAYV infection at 7 days PI. (D) MAYV infection at 10 days PI. (E) MAYV infection at 15 days PI. (F) MAYV infection at 20 days PI, with centralized nuclei (arrow). (G) Histological score. Images (200X magnification, bar 40 μm) N = 3 animals/group.

**Fig 9:**
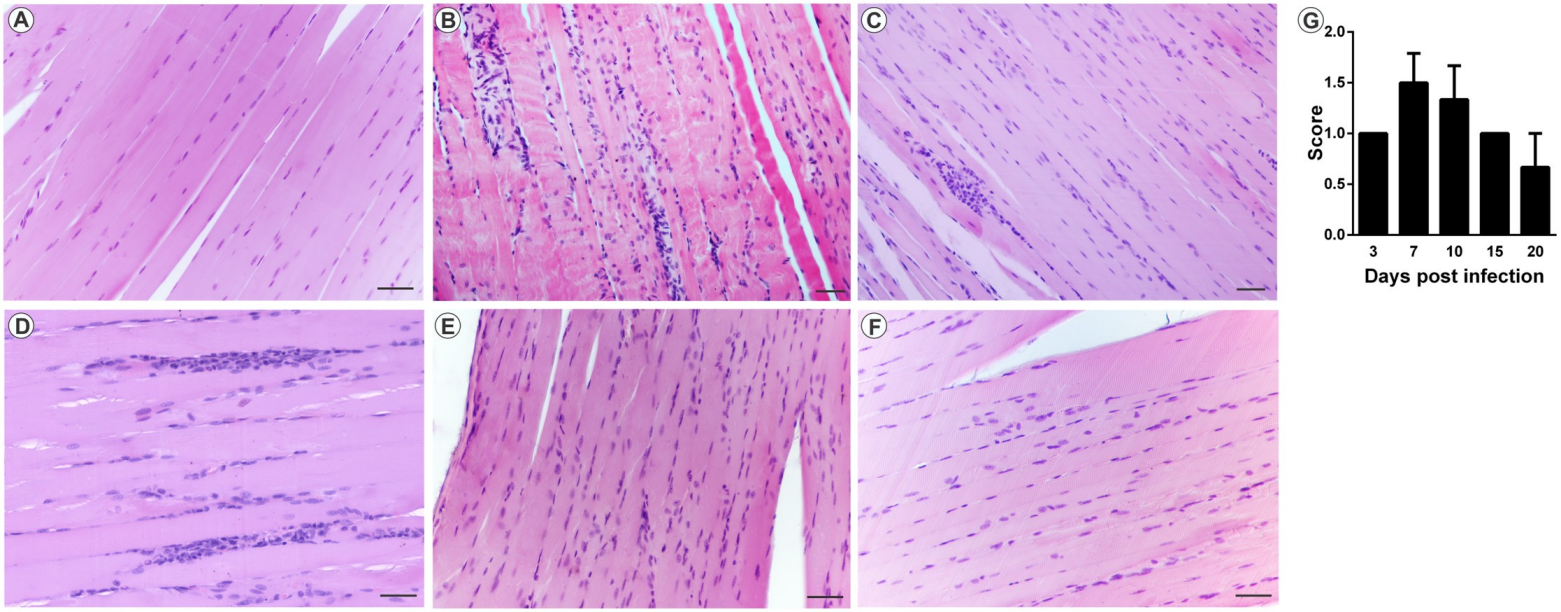
Mayaro virus-induced myositis in the contralateral muscle in mice infected via the rear left footpad with 2.57 x 10^6^ PFU. The animals were sacrificed on days 3, 7, 10, 15 and 20 PI. for tissue damage analysis. The quadricep muscles was fixed in Karnovsky, embedded in paraffin, and 5μm sections were made and stained with HE. (A) Negative control. (B) MAYV infection at 3 days PI. (C) MAYV infection at 7 days PI. (D) MAYV infection at 10 days PI. (E) MAYV infection at 15 days PI. (F) MAYV infection at 20 days PI. (G) Histological score. Images (200X magnification, 40 μm bar). N = 3 animals/group.

The analysis of MAYV-induced morphological changes was performed on skeletal muscles and joints, on 3, 7, 10, 15 and 20 days PI. Myositis occurred symmetrically for both infection pathways, affecting the skeletal muscle of the pelvic limbs in a similar way. In FLP, little or no inflammation was observed on day 3 PI, with the muscle tissue being very similar to the control (Figs 5B and 6B). However, on days 7 and 10 PI, an increased number of inflammatory infiltrates were observed (Fig 5C, 5D and 5J; Fig 6C, 6D and 6J), concentrated in specific points, but disperse in all tissue. Additionally, there is also the presence of degenerating fibers (Fig 5C, 5D, 5G and 5H; Fig 6C and 6H), which are characterized by a pale color, swollen appearance and central nucleus. Collagen deposition (Fig 5C) and presence of vasculitis were also observed (Fig 5I). With 15 and 20 days PI there were still small foci of inflammatory infiltrate. In the joints and bones of the foot, inflammation started on day 3 PI, and the presence of inflammatory cells in the synovium, tendon, ligament, periosteum and extensive inflammation and destruction of associated muscle was observed (Fig 7). In FPP, the inflammation in the muscle began on day 3 PI, with the presence of degenerating fibers (Figs 8B and 9B). On days 7 and 10, the inflammatory process continued (Figs 8C and 8D, 9C and 9D), but there was no difference in the histological score between post-infection times (Figs 8G and 9G). At day 15 PI there were still few infiltrate cells (Figs 8E and 9E), with full tissue recovery on day 20 PI (Figs 8F and 9F). In both groups, tissue recovery occurred between 15 and 20 days PI, which is indicated by the presence of fibers with rows of centralized nuclei (Figs 5E, 6H and 8F).

### Cytokine profile assessment

**Fig 10:**
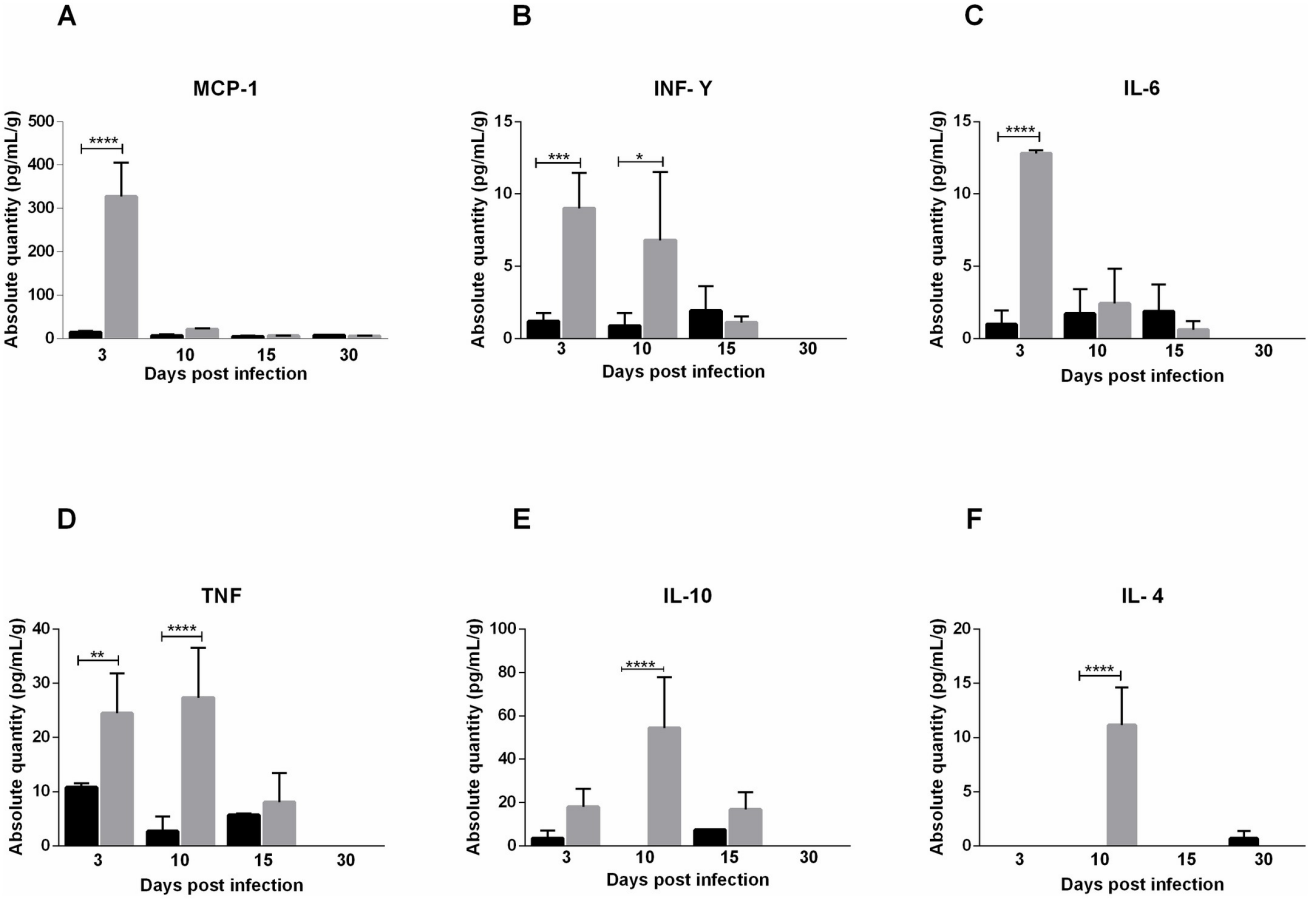
Cytokine profile of 15-day old Balb/c mice infected below the forelimb with 1.25x10^7^ PFU. Serum was collected 3, 10, 15 and 30 days post-infection for analysis of cytokine levels. At day 3 PI levels of MCP-1 (A), TNF (B), INF-γ (C) and IL-6 (D) were high, and TNF (B) and INF-γ levels up to the 10th day. IL-10 (E) and IL-4 (F) levels increased at day 10 PI. Statistics were performed with one-way ANOVA, Bonferroni’s multiple comparisons test. Gray bars: Infected. Black bars: negative control. (* P value ≤ 0.05; ** P value ≤ 0.01; *** P value ≤ 0.001; **** P value ≤ 0.0001). N = 3 animals/group.

**Fig 11:**
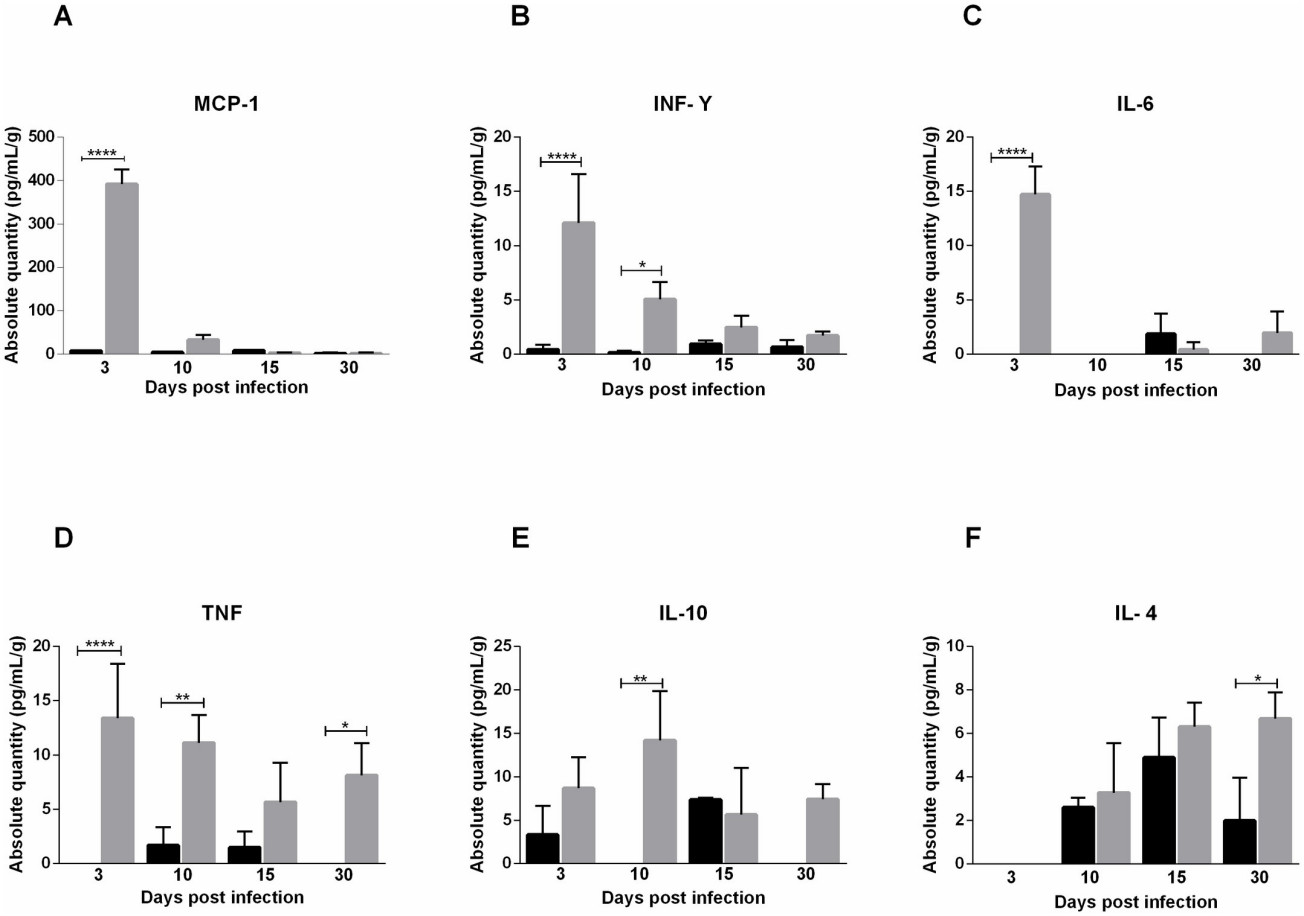
Cytokine profile of 15-day old Balb/c mice infected via the rear footpad with 2.57 x 10^6^ PFU. Serum was collected 3, 10, 15 and 30 days post-infection for analysis of soluble factor levels. The levels of MCP-1 (A), TNF (B), INF-γ (C) and IL-6 (D) were elevated on day 3 PI. At day 10, TNF (B) and INF-γ (C) remained high and the level of IL-10 (E) was elevated. At day 30 PI TNF (B) and IL-4 (F) were present. Statistics were performed with one-way ANOVA, Bonferroni’s multiple comparisons test. Gray bars: Infected. Black bars: negative control. (* P value ≤ 0.05; ** P value ≤ 0.01; **** P value ≤ 0.0001). N = 3 animals/group.

**Fig 12:**
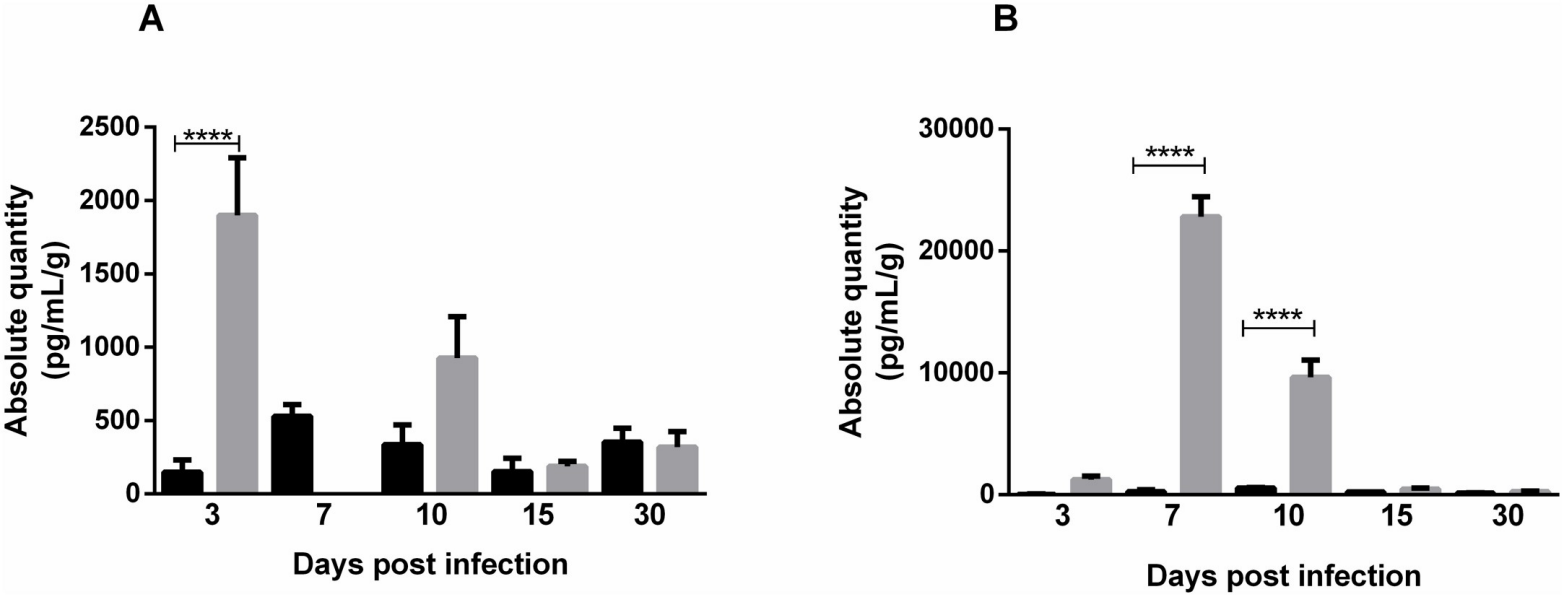
Detection of MCP-1 in muscle tissue. In the group infected below the forelimb (1.25x10^7^ PFU) MCP-1 was elevated only on day 3PI (A). While in the group infected via the rear footpad (2.57x10^6^ PFU), MCP-1 was detected on days 7 and 10PI (B). Gray bars: Infected. Black bars: negative control. Statistics were performed with one-way ANOVA, Bonferroni’s multiple comparisons test. (**** P value ≤ 0.0001). N = 3 animals/group.

MAYV infection initially activates the Th1 pattern, inducing a strong inflammatory response, which is followed by cytokine induction of the Th2 response. Both groups showed similar cytokine profile in serum. On the third day after infection, high levels of proinflammatory cytokines TNF, INF-γ and IL-6 and chemokine MCP-1 were detected in the serum of both infected groups. TNF and INF-γ levels remained high until day 10, when an increase in the anti-inflammatory cytokine IL-10 was also observed. In FPP at 30 days PI, IL-4 and TNF were detected again. In FLP, the IL-4 cytokine was present at 10 days PI and on 30 days PI no cytokines were detected (Figs 10 and 11). In muscle tissue, MCP-1 was detected at high levels at 3 days PI in FLP (Fig 12A), while in FPP it was present on days 7 and 10 PI (Fig 12B). Cytokines IL-9, IL-13 and IL-12p70 were not found in either serum or muscle tissue.

### MAYV replication kinetics

**Fig 13:**
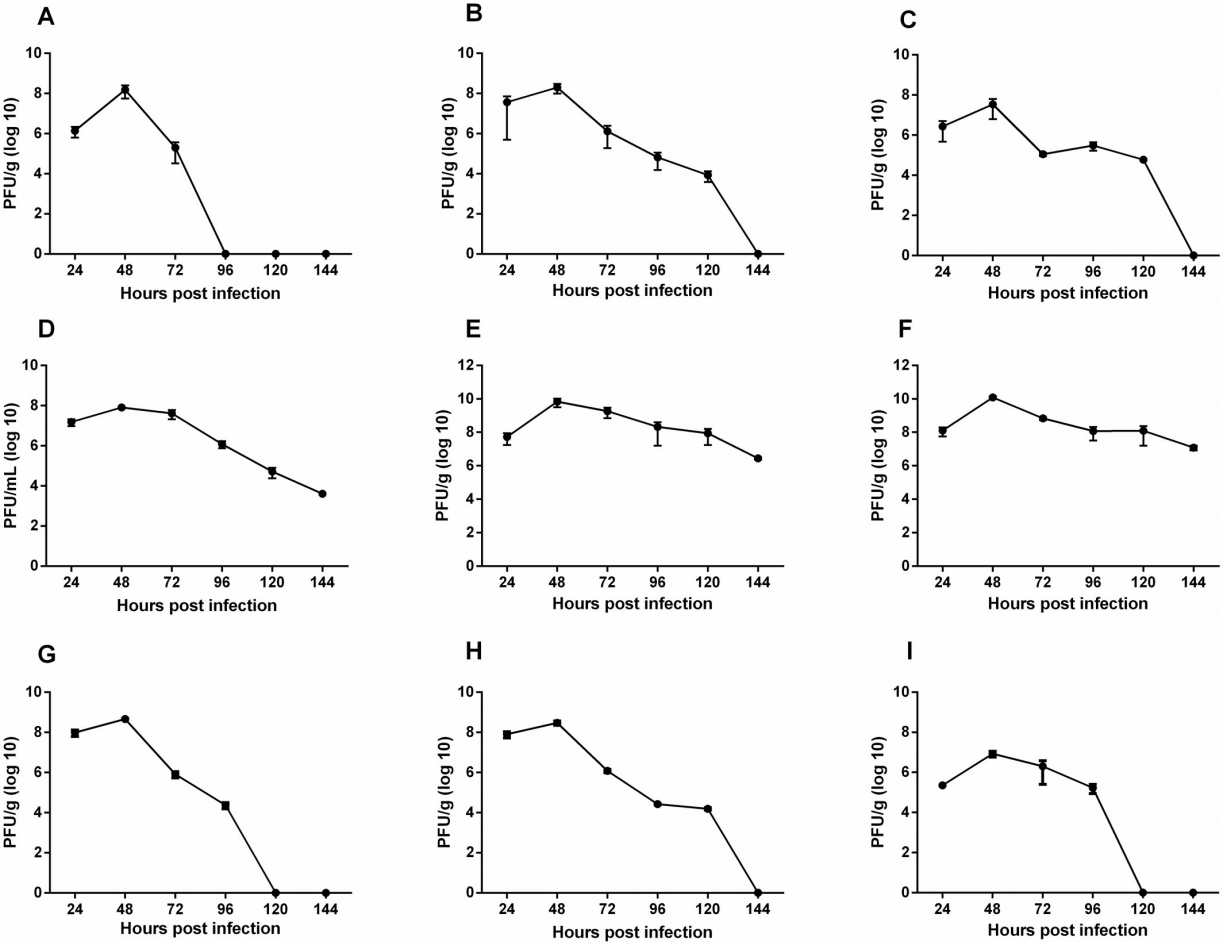
MAYV load on organ and tissues. 15-day old Balb/c mice were infected below the forelimb with 1.25x10^7^ PFU. At 24, 48, 72, 96, 120 and 144 hpi, liver (A), spleen (B), heart (C), serum (D), right and left ankles E and F), right and left quadricep muscles (G and H) and eyes (I) were harvested and homogenized, and the amount of infectious virus present was quantified by plaque assay on Vero cells. N = 3 animals/group.

**Fig 14:**
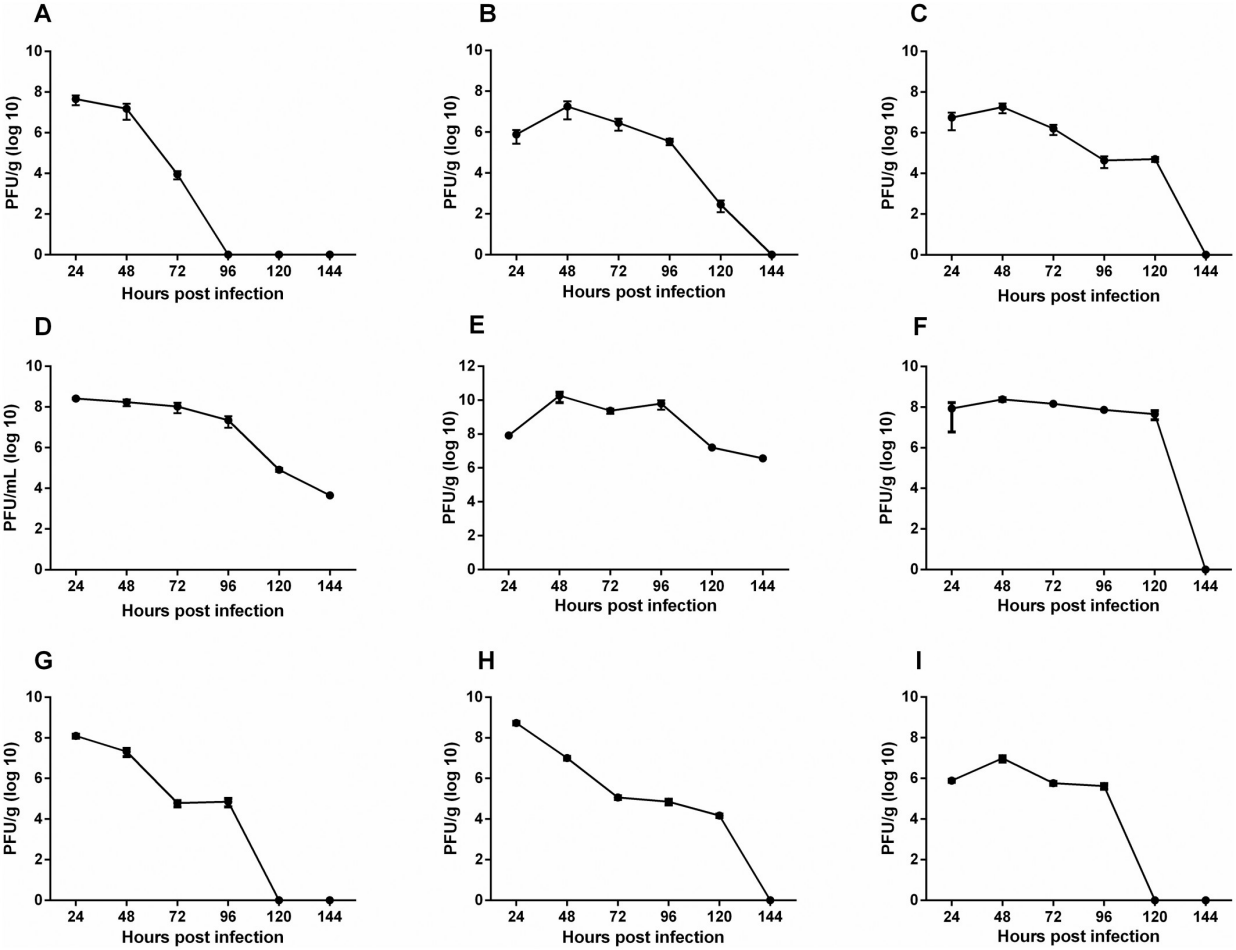
MAYV load on organ and tissues. 15-day old Balb/c mice infected in the rear footpad with 2.57 x 10^6^ PFU. At 24, 48, 72, 96, 120 and 144 hpi, liver (A), spleen (B), heart (C), serum (D), right and left ankles E and F), right and left quadricep muscles (G and H) and eyes (I) were harvested and homogenized, and the amount of infectious virus present was quantified by plaque assay on Vero cells. N = 3 animals/group.

Peak titers of infectious virus were detected at 24 to 48 hours post-infection (hpi) in both infection pathways (Figs 13 and 14). In animals from FLP the highest viral titers were detected in the left and right ankles with 10^9^ PFU/g and 10^10^ PFU/g, respectively (Fig 13E and 13F), and live, spleen and muscles reached a peak of 10^8^ PFU/g (Fig 13A, 13B, 13G and 13H). In FPP animals, viral titers of up to 10^10^ PFU/g were detected in the right ankle (Fig 14D), and 10^8^ PFU/g in the muscles and left ankle (Fig 14F, 14G and 14H). MAYV has also been found in other organs such as the heart and eye. The viremia persisted for up to 144 hpi in both groups.

### Pain assessment

**Fig 15:**
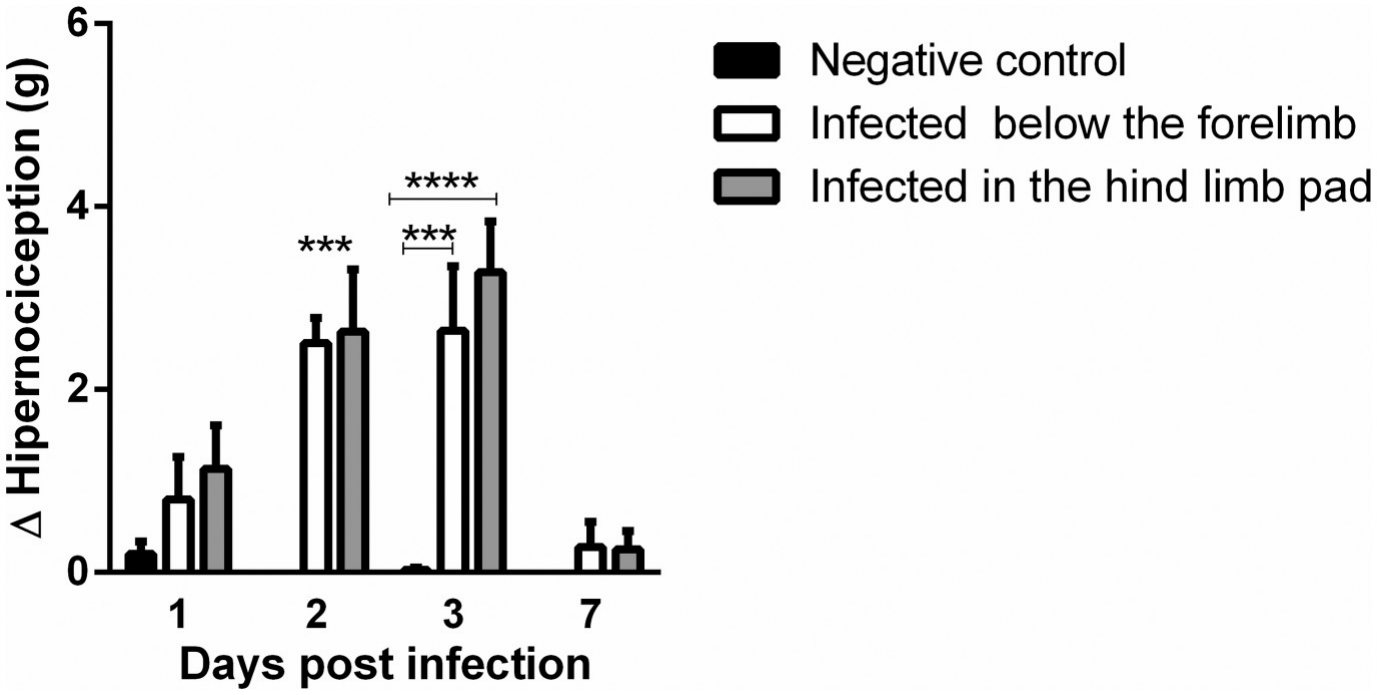
Evaluation of the pain threshold by the von Frey method in animals infected with the Mayaro virus. Statistics were performed with two-way ANOVA, Tukey’s multiple comparisons test (*** P value ≤ 0.001; **** P value ≤ 0.0001). N = 8 animals/group.

Until now, we have shown that MAYV infection induced clinical, morphological and inflammatory changes. Our next question was if these inflammatory indices were associated with articular dysfunction. For that, mice with 28 days were used to evaluate the intensity of hypernociception. Our results revealed that at 2 and 3 days PI, both mice from FLP and FPP groups, showed a decrease in their nociceptive thresholds in comparison to MOCK-infected groups. From day 7 PI the nociceptive thresholds were already similar to baseline levels (Fig 15). These animals did not show any sign of disease besides pain.

## Discussion

The arthritogenic alphaviruses include chikungunya (CHIKV), Sindbis (SINV), Ross River (RRV), Mayaro (MAYV), Barmah Forest (BFV) and O′nyong′nyong (ONNV) viruses, which are associated with arthritis and/or debilitating arthralgia, and in some cases with bone pathologies [14,15]. These viruses are considered an important socioeconomic problem, since they have the potential to cause large epidemics with a significant impact on human health and the economy, due to their debilitating effects [16]. The current understanding of the mechanisms involved in alphaviral pathogenesis has been based on studies with animal models and clinical observations, mainly with RRV and CHIKV. With the risk of emergence and urbanization of the MAYV in South America and other continents, it has become necessary to understand the pathology and immune response of this virus. Therefore, this research presents an animal model of arthritis and myositis induced by the MAYV.

In the identification of murine as animal models for alphavirus, the age of the animal has been considered a determining factor in the susceptibility and severity of the disease. As neonatal and young mice are more susceptible, they have been the most used to reproduce alphaviral disease [17,18]. The resistance of adult mice to alphavirus infection is due to type I interferons, which quickly provide an antiviral state [17,19]. It has been shown in experiments with adult IFN-α/βR^+/-^ and IFN-α/βR^-/-^ mice that develop moderate or severe disease, respectively [19]. Over time several animal models for arthritogenic alphaviruses have been tested in different strains of neonatal or young mice, including CD-1, Swiss, Balb/c and C57BL/6 [17]. In the present study, it was shown that the Balb/c mice at 15 days old and, regardless of the infection pathway, were susceptible to the disease induced by MAYV.

Clinical signs such as ruffled fur, hunched posture and eye irritation were common and frequent in both infection pathways. Eye irritation is a frequent symptom in patients with Mayaro fever, who report photophobia and retroocular pain [5,13]. This clinical sign in the animal model was described only for sindbis virus, but the infection pathway used was intracranial, a localized infection pathway [20]. However, in other animal models for alphavirus, such as CHIKV and RRV, which also used peripheral infection pathways, no eye irritation was reported [18]. In addition, it has been reported that CHIKV can infect humans’ cornea and be transmitted by the ocular pathway in animals [21], and here we demonstrate that the MAYV is able to replicate in the eye of the animals. Therefore, it is important to investigate their potential for transmission by this pathway. Signs of disease such as gait alteration, weight loss and death occurred only in animals infected below the forelimb and have been described for pathway-independent RRV [18]. Edema in paw, which is a characteristic sign of alphavirus infection, has also been reported for MAYV in models using the C57BL / 6 and A129 lines, however, it was not observed in this work [22,23]. As for the signs of tremors, hunched posture and animals falling and circulating spontaneously, no reports were found in the literature. The kinetics of MAYV replication showed a similar pattern in the two infection pathways studied, with the virus persisting longer in the joints than in the other tissues. In addition, both pathways were efficient at spreading the virus. The presence of alphavirus in the heart and eye in other animal models was not reported in the literature, making it necessary to investigate the tropisms of other alphaviruses by these tissues.

The infection also resulted in several histopathological changes, such as myositis, tenosynovitis, synovitis and periostitis. These tissue changes were responsible for the lower strength of infected animals in the physical test. This resistance loss was more intense between 10 and 15 days PI, corresponding to the tissue damage and the beginning of the regeneration process. Both infection pathways could cause myositis, which was characterized by presence of infiltrates and destruction of muscle fibers. Tissue damage described for MAYV is similar to that found in animal models for CHIKV and RRV, with extensive degeneration and necrosis of skeletal muscle, inflammation of the periosteum and tissues associated with the joint being reported [18,24].

New, potentially aggressive lesions have been identified in CHIKV infections in animals, such as necrosis of cartilage and bone proliferation in the periosteum. In patients with CHIKV, imaging studies have revealed the presence of tenosynovitis, synovial thickening, periostitis, periosteum proliferation and bone erosion. These clinical findings show that the initial acute disease may progress to chronic erosive arthritis [15,24]. In this study, identification of tenosynovitis and periostitis in animals infected by MAYV emphasizes the importance of imaging tests in clinical studies of patients with prolonged polyarthralgia.

As with other alphavirus infections, Mayaro fever causes persistent, often debilitating joint pain [25]. Joint pain is due to the action of inflammatory cytokines, such as IL-6, TNF-α and IL-1β, as well as tissue damage, which sensitizes nociceptors [26]. The presence of pain at only 2 and 3 days PI shows that induced-MAYV pain is associated with the inflammatory response, which is characterized by elevated IL-6 and TNF-α levels on day 3 PI.

The evaluation of the cytokine profile showed that MAYV induces a strong inflammatory response in early days of infection, mediated by cytokines TNF-α, IL-6 and INF-γ and chemokine MCP-1. These mediators have also been expressed in animal models for RRV and CHIKV, and more recently it has been demonstrated in patients with Mayaro Fever [27,28]. High levels of IL-6 and TNF-α during early stages are probably responsible for inducing pain observed in animals on day 3 PI. In patients with chronic arthralgia in MAYV infection, these factors are also elevated [28]. Initial inflammatory response in animals was rapidly controlled by anti-inflammatory action of IL-4 and IL-10, and in later stages by IL-4. Besides the control of immune response, IL-10 also plays an important role in muscle regeneration by inducing the exchange of macrophages from the M1 to M2 phenotype in the injured muscle [29]. The action of IL-10 on tissue recovery is indicated by the presence of fibers with centralized nuclei, which is a characteristic of the muscle regeneration process. The presence of INF-γ and IL-4 suggests the participation of adaptive immunity. A second increase in TNF-α levels in animals FPP after 30 PI may indicate the persistence of viral RNA in the tissue, as has been demonstrated for CHIKV [30].

Patients with prolonged arthralgia during MAYV infection show that levels of IL-1Ra, IL-6, IL-7, IL-8, IL-13, IL-17, G-CSF, IFN-γ, PDGF- α, VEGF and IL-12p70 remained elevated up to 12 months after infection. The acute phase was marked by a high expression of MCP-1, while IL-9 and IL-2 were predominant during the convalescent phase. These data showed that a robust early inflammatory response is associated with the development of chronic arthralgia [28]. Cytokines IL-13, IL-9, IL-12p70 and IL-17 were also evaluated in animals but showed no differences in relation to the control. The major anti-inflammatory mediators induced in humans were IL-1Rα and PDGF-BB [28], different to the results found in this study using animals, in which IL-10 was the major agent responsible for inflammation control.

These results show that the animal model for Mayaro virus has been able to reproduce the acute disease that occurs in humans, regardless of the infection pathway, and is adequate to understand most events resulting from MAYV infection, such as tissue damage and inflammatory response. The presence of the cytokines MCP-1, TNF and INF-γ serum, as well as MCP-1 in the muscle, indicate the action of macrophages in target tissues of infection, which makes this cell and MCP-1 important elements common in therapy of arthritis caused by RRV, CHIKV and MAYV [31,32]. It is important that further studies are conducted to evaluate the role of these immune factors in the pathogenesis induced MAYV. Additional efforts need to be made in cohort studies to obtain more information about the course of the disease in humans, since we presented evidence here for ocular infection and severe tissue damage.

## Materials and methods

### Ethics statement

Animals were anesthesiaded with ketamine/Xylazine in all procedures and all experiments were done with agreement of Ethics Committee on Animal Use (Comitê de Ética no Uso de Animais—CEUA) of the Federal University of Viçosa, protocol number 63–2013, established by the National Animal Experimentation Control Council (Conselho Nacional de Controle de Experimentação Animal—CONCEA), an organ that is part of the Brazilian Ministry of Science, Technology and Innovation (MCTI).

### Virus titration

The Mayaro virus (ATCC VR-66, strain TR 4675) was kindly provided by Dr. Davis Ferreira, Federal University of Rio de Janeiro—UFRJ. This strain corresponds to the first Mayaro virus isolate in Trinidad, 1954. The passages history of this strain, includes passages in the mouse brain [33], and the MAYV stock used in this work was propagated in C636 cells whenever necessary for viral titre increase. Vero cells were propagated at 37 °C in minimum essential media (MEM) supplemented with 10% fetal bovine serum (FBS), which were selected and plated in transfer to 24-well plate (3 X 10^5^ cells per well) and used for virus titrations. The plates were incubated at 37 °C and, upon reaching ∼80% confluence, the medium MEM was removed. 100μl of each 10-fold virus dilution was then added, and the control wells received phosphate-buffered saline (PBS). After 1 hour incubated in the rotary shaker, 1.5 mL of CMC 3% (carboxymethylcellulose 3%, supplemented with 2% FBS) was added. Plates were returned to incubation at 37 °C for more 48 h. Throughout this time, cytopathic effects were observed. After incubation, 500μL of CMC 3% from each well was removed, 1 mL of formaldehyde 20% was added and maintained for 1 hour under these incubation conditions. Subsequently, the plates were washed and 500μL of 5% crystal violet (CV) was added. The plates were incubated for 30 minutes with the dye. After incubation, the plates were washed for lysis plate count. To determine viral titers in tissues, mice were sacrificed by exsanguination and perfused with 1X PBS. The right and left ankles, right and left quadricep muscles, spleen, liver, eyes and heart were removed by dissection at 24, 48, 72, 96, 120 and 144 hours post-infection. The tissues were weighed and then homogenized in MEM media supplemented with 10% FBS and stored at 80°C until viral load. To perform a 10X serial dilution after maceration of the tissue, the weight of each tissue was multiplied by nine, and the final value corresponds to the volume of medium required for each tissue, weight of organ as 10% of the total volume. All experiments were performed 5 times.

### Mice

Balb/c mice obtained from the animal facility of the Federal University of Viçosa (Central Bioterium), with agreement of Ethics Committee on Animal Use (Comitê de Ética no Uso de Animais—CEUA) of the Federal University of Viçosa, protocol number 63–2013. The 15 day-old, female and male mice were subcutaneously inoculated with high doses of MAYV in the right rear footpad and in the thorax, below the right forelimb. The animals infected in the thorax, below the right forelimb (FLP) received 50 μL (1.25 x 10^7^ PFU) of viral suspension, while the animals infected in the right rear footpad (FPP) received 10 μL (2.57x10^6^ PFU) of virus suspension in PBS. Mock-infected mice received the PBS diluent alone. Cohort: 12/group for evaluation of clinical signs and physical test; 8/group for evaluation of pain; 3/group for evaluation of tissue damage and cytokine profile.

### Evaluation of clinical signs and physical test

Animals were observed for a period of 21 days. During this time, they were monitored every 24 hours for the appearance of signs of disease, such as weight loss and muscle weakness. The physical test used to evaluate muscle weakness was wire-hanging, modified from the method described by Sango, McDonald [34]. This method uses a metal grid and a box lined with wood shavings; the mice are placed on the grid and it is shaken to make the animal hold on. The grid is then inverted, placed over the box and maintained at a height of 23 cm. The tendency to fall is measured using a stopwatch, and a maximum time of 2 minutes is established.

### Cytometric bead array

At 3, 10, 15 and 30 days post-infection, the serum was collected, centrifuged at 514xg for 10 minutes and stored at -20 °C. The levels of serum cytokines were determined using the CBA mouse Th1/Th2/Th17 kit (BD Biosciences), which simultaneously quantifies IL-2, IL-4, IL-6, IL-10, IL-17A, IFN-γ and TNF and CBA flex IL-9, IL-13, MCP-1 and IL12-p70, following manufacturer′s instructions. The level of cytokines was also evaluated in muscle tissue at 3, 7, 10, 15 and 30 days post-infection. For this, the muscle was macerated in PBS, centrifuged at 514 xg and stored at -80 °C. The data were acquired using by Flow cytometer BD FACS Verse.

### Histological analysis

For histology, the mice were sacrificed and the quadricep muscles and ankle joints were removed and fixed in Karnovsky fixative (paraformaldehyde 4% and glutaraldehyde 4%, pH 7.3). The bone-associated tissues were decalcified with EDTA 10%, pH 7. Further, the tissues were embedded in paraffin and a 5 μm section obtained by microtomy, stained with hematoxylin and eosin (H & E) and observed by light microscopy. The quantification of the inflammatory cell infiltration was performed based on the following score: 0 without infiltrates; 1 light infiltrates; 2 moderate infiltrates; 3 severe infiltrates. The score considers the number of infiltrates, the size and their distribution in the tissue. For each time the best slide of each animal (N = 3) was chosen to quantify the inflammatory infiltrate. After analysis the scores were added.

### Evaluation of mechanical hypernociception by the von Frey method

The electronic method of von Frey is based on the use of an apparatus (anesthesiometer electronic) that has a pressure transducer connected to a digital force counter expressed in grams (g). The pressure transducer is adapted to a polypropylene tip, which is brought into contact with the animal′s leg [35]. Nociceptive thresholds were determined by exerting a linearly increasing pressure on the center of the animal′s paw until a flexion reflex occurred, followed by a flinch response upon withdrawal of the paw. The pressure-meter automatically records the pressure value when the foot is removed. To perform the test, the animals were placed in acrylic boxes with non-malleable wire floor for 15 minutes for aclimatization. A sloping mirror below the railing provides a clear view of the animal hindpaw during measurements. The baseline measurements of all groups were performed before infection, and the other measurements were taken at 3, 7, 10, 15 and 21 days postinfection. There were always two measures per animal at a time. The intensity of hypernociception was quantified by the difference between baseline and the other evaluated times (delta = basal—other times). In this experiment, animals with 28 days of age were used, since it is the minimum age required to perform the test.
